# Feasibility of single-site laparoscopic colectomy with complete mesocolic excision for colon cancer: a prospective case–control comparison

**DOI:** 10.1007/s00464-013-3284-x

**Published:** 2013-11-08

**Authors:** Ichiro Takemasa, Mamoru Uemura, Junichi Nishimura, Tsunekazu Mizushima, Hirofumi Yamamoto, Masataka Ikeda, Mitsugu Sekimoto, Yuichiro Doki, Masaki Mori

**Affiliations:** 1 Department of Gastroenterological Surgery, Graduate School of Medicine, Osaka University, 2-2 Yamadaoka, Suita, Osaka 565-0871 Japan; 2 Department of Surgery, Osaka National Hospital, 2-1-14 Hoenzaka, Chuoku, Osaka 540-0006 Japan

**Keywords:** Single-site laparoscopy colectomy, Complete mesocolic excision, Short-term outcome, Oncologic clearance, Colon cancer

## Abstract

**Background:**

Single-site laparoscopic colectomy (SLC) is an emerging concept that, compared with conventional multiport laparoscopic colectomy (MLC), yields reduced postoperative pain and improved cosmesis. Complete mesocolic excision (CME) is a novel concept for colon cancer surgery that provides improved oncologic outcomes; however, there are no reports of SLC with CME. We conducted a prospective case–control study to evaluate the feasibility and safety of SLC with CME for colon cancer.

**Methods:**

Prospectively collected data of patients with stage I-III colon cancer who underwent SLC (*n* = 150) or MLC (*n* = 150) between June 2008 and March 2012 were analyzed. Patients who underwent SLC were, in terms of clinical characteristics and tumor location, matched as closely as possible with those undergoing MLC. Within each group, patients were classified as having right-sided (*n* = 69 in each group) or left-sided (*n* = 81 in each group) colon cancer, and short-term outcomes were compared between the two procedures overall and per side.

**Results:**

Overall perioperative outcomes, including operation time, blood loss, number of lymph nodes harvested, length of the resected specimen, and complications, were similar between the two procedures, whereas postoperative pain was significantly lower with SLC. Operation time for right-sided SLC was significantly shortened. SLC with CME was completed successfully in 94 % (65/69) of right-sided cases and in 88 % (71/81) of left-sided cases. Conversion rates were 1.4 % (1/69) and 1.1 % (1/81), respectively. The umbilical scars were nearly invisible 3 months after the procedure, and most patients reported being quite satisfied with the cosmetic outcomes.

**Conclusions:**

SLC with CME for colon cancer is feasible when performed by experienced surgeons in selected patients. Excellent cosmesis and reduced postoperative pain as well as oncologic clearance can be expected. A large-scale, prospective, randomized, controlled trial should be conducted to confirm the superiority of this procedure over MLC with CME.

Laparoscopic surgery plays a central role as a meaningful option in the management of colon cancer [1]. Laparoscopic colectomy has been compared to open colectomy in several multicenter, prospective, randomized, controlled trials (RCTs), and the short-term advantages and similar long-term survival achieved with laparoscopic colectomy have been well established by [2–5].

Complete mesocolic excision (CME) with central vascular ligation (CVL), according to the sound principles of total mesorectal excision (TME) [6, 7] for rectal cancer, has been translated to colon cancer under the concept of radical oncologic resection and following embryologic tissue planes along with the entire regional mesocolon in an intact fascial coverage of the tumor and its lymphatic drainage, including a high arterial tie [8, 9]. Data suggest that CME with CVL maximizes lymph node harvest, which may lead to improved oncologic outcomes [9, 10]. The technical feasibility and safety of laparoscopic CME for colon cancer also has been reported [11, 12].

Single-site laparoscopic colectomy (SLC) is performed entirely through one extraction site, theoretically reducing postoperative pain and the risk of abdominal wall morbidities, including bleeding, hernia, and internal organ damage, whereas conventional multiport laparoscopic colectomy (MLC) requires several ports and abdominal incisions [13]. Current efforts in minimally invasive treatment have shifted toward decreasing trauma by reducing the number of ports and/or size of the trocars [14]. Several groups have reported the feasibility and benefits of SLC, including improved cosmesis, reduced postoperative pain, and shortened recovery time, but there are some limitations including technical problems, such as instrument crowding, in-line viewing, insufficient countertraction, somewhat narrow patient applicability, and increased costs [15–22]. In addition, concerns over oncologic clearance in SLC remain unsettled. The less invasive procedure may bring patients some happiness or satisfaction, but oncologic clearance and technical safety are of utmost importance in the surgical treatment of colon cancer. We believe that CME also is effective and important in this minimally invasive procedure for colon cancer, especially for a locally advanced lesion; however, there is no report of SLC with CME for colon cancer at present. Therefore, we conducted a study to evaluate the feasibility and safety of SLC with CME for colon cancer in a prospective case–control analysis that examined short-term surgical results.

## Patients and methods

### Patients and data collection

We identified all patients scheduled to undergo SLC between 2008 and March 2012. The SLCs included right hemicolectomy for cancer of the cecum or ascending colon (right-sided colon cancer), and left hemicolectomy, sigmoidectomy, and anterior resection for cancer of the descending, sigmoid, or rectosigmoid colon (left-sided colon cancer).

In total, 150 patients undergoing SLC and 150 patients undergoing MLC during the same period and matched as closely as possible to the SLC patients were included in the study. Age, sex, body mass index (BMI), American Society of Anesthesiologists (ASA) class, tumor location, tumor size, preoperative disease stage, personal history of prior surgery, operation time, estimated blood loss, length of the incision (initial length and length required for extraction), number of lymph nodes harvested, length of the resected specimen, conversion to open surgery, insertion of an additional port, perioperative complications, morbidity, pain on postoperative day (POD) 1 (as indicated by the patient on a visual analog scale (VAS), and length of hospital stay were recorded. Patient characteristics are shown in total, per treatment group, and per right- versus left-sided procedure in Table 1.

**Table 1:** Patient characteristics

	SLC-total (*n* = 150)	MLC-total (*n* = 150)		SLC-R (*n* = 69)	MLC-R (*n* = 69)		SLC-L (*n* = 81)	MLC-L (*n* = 81)	
			*p* value			*p* value			*p* value
Age (year)	64.3 ± 11.7	65.6 ± 12.5	0.353	65.0 ± 11.8	66.6 ± 11.9	0.425	64.3 ± 11.7	64.8 ± 13.0	0.797
Sex (male/female)	75/75	71/79	0.644	31/38	36/33	0.394	37/44	35/46	0.752
BMI (kg/m^2^)	21.7 ± 3.3	22.4 ± 4.7	0.137	21.5 ± 3.5	22.2 ± 3.7	0.257	21.9 ± 3.3	22.7 ± 5.4	0.257
ASA physical status									
1	40	33	0.572	18	15	0.807	22	18	0.704
2	83	85		38	39		45	46	
3	27	32		13	15		14	17	
Tumor location									
Cecum	34	29	0.440	34	29	0.393			
Ascending colon	35	40		35	40				
Descending colon	6	9					6	9	0.414
Sigmoid colon	53	45					53	45	
Rectosigmoid colon	22	32					22	27	
Preoperative disease stage									
I	76	65	0.290	32	31	0.82	44	34	0.220
II	48	49		23	21		25	28	
III	26	36		14	17		12	19	
Prior surgery (%)	31 (21)	39 (26)	0.275	16 (23)	19 (27)	0.557	15 (19)	20 (25)	0.340

Number (and percentage) of cases are shown unless otherwise indicated

*SLC* single site laparoscopic colectomy, *MLC* multiport laparoscopic colectomy, *BMI* body mass index, *ASA* American Society of Anesthesiologists, *L* left, *R* right

The criteria for SLC were as follows: stage I-III colon cancer, tumor diameter <4 cm, body mass index (BMI) <35 kg/m^2^, and ASA physical status <2. Each SLC patient was matched for clinical characteristics (age, sex, BMI, preoperative disease stage, prior surgery) and location of the tumor (right side of the colon or left side of the colon) to a patient undergoing MLC. No patient with rectal cancer, an advanced T4 tumor, a huge or bulky tumor ≥4 cm, severe obesity, perforated tumor, stenosis with bowel distention, prior abdominal polysurgery, or any severe comorbidity was included in the study. Patients in both groups were subclassified as those with right-sided colon cancer (*n* = 69 in each group) and those with left-sided colon cancer (*n* = 81 in each group).

### Surgical techniques

All SLCs with CME were performed by one of two well-experienced laparoscopic colorectal surgeons who followed similar techniques. The conventional MLCs with CME were performed by one of five laparoscopic colorectal surgeons including the two well-experienced surgeons.

The entire SLC procedure was performed with standard laparoscopic instruments through an initial 2- to 3-cm extraction incision in the umbilicus [13]. A multichannel access device, such as a SILS Port (Covidien, Mansfield, MA, USA) or EZ Access (Hakko, Nagano, Japan), was fitted into the incision and rotated to achieve the ideal operative view and triangulation and to avoid or resolve collision of the instruments. An additional incision or trocar port was placed without hesitation if necessary to complete the procedure, and conversion to open laparotomy was maintained as an option. The indication and timing of trocar insertion or conversion to open surgery depended on the surgeon’s judgment.

The abdominal cavity was explored with a 30-degree, 10-mm rigid laparoscope in all patients, with CO_2_ pneumoperitoneum established and maintained at 10 mmHg. Conventional MLC required five ports, with the first 12-mm trocar in the umbilicus as a camera port, another 12-mm trocar, and three 5-mm trocars. The trocars were inserted at the right and left, upper and lower abdominal quadrant under laparoscopic guidance. The camera port was expanded to extract the specimen through an incision of 2–5 cm, as previously described [2–5].

**Fig. 1:**
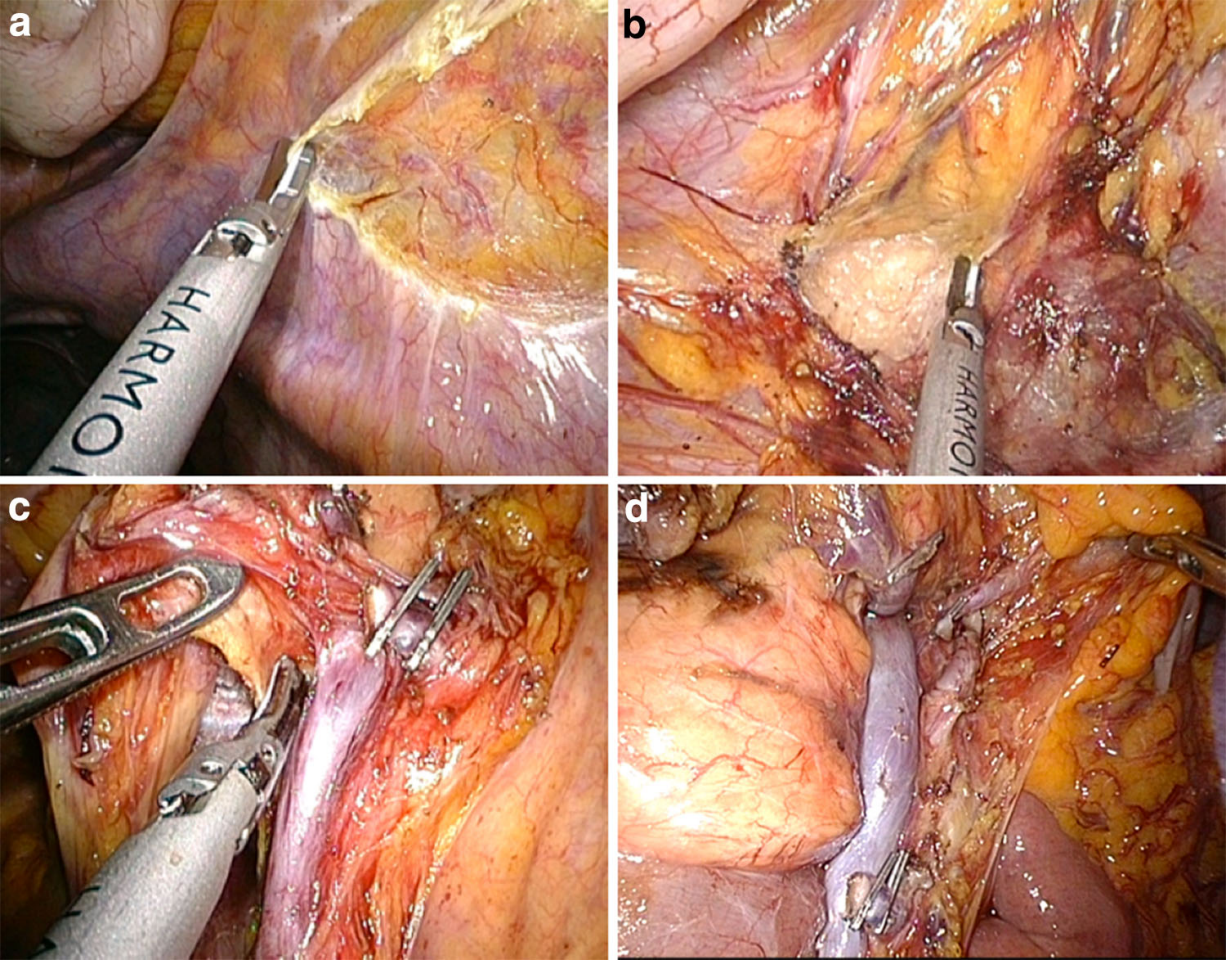
Operative techniques for single-site laparoscopic right hemicolectomy with complete mesocolic excision for ascending colon cancer. **A** Inferior approach with initial peritoneal dissection between the mesoileum and the retroperitoneum. **B** Exposure of the head of the pancreas and mobilization of the duodenum by complete mesocolic excision. **C** Ligation at the origin of the ileocolic artery and vein with dissection of the entire the right-side mesocolon. **D** Completion of the lymphadenectomy in complete mesocolic excision with central vascular ligation for ascending colon cancer

Right hemicolectomy for right-sided colon cancer in both groups was performed via an inferior approach, with initial peritoneal dissection between the mesoileum and the retroperitoneum performed with the patient in the Trendelenburg position (Fig. 1A). After intact mesocolic plane resection by CME, the duodenum and pancreas were sufficiently exposed (Fig. 1B), and the ileocolic vessels were ligated and dissected between clips at their origin to allow dissection of the entire right mesocolon (Fig. 1C). Laparoscopic CME with CVL was completed by dissecting the lymph nodes and lymphatic tissues at the origin of the ileocolic, right colic, and middle colic vessels (Fig. 1D). After dissection of the greater omentum, the hepatic flexure was mobilized. The specimen was extracted through the minilaparotomy incision in the umbilicus, after which extracorporeal functional end-to-end anastomosis was performed.

**Fig. 2:**
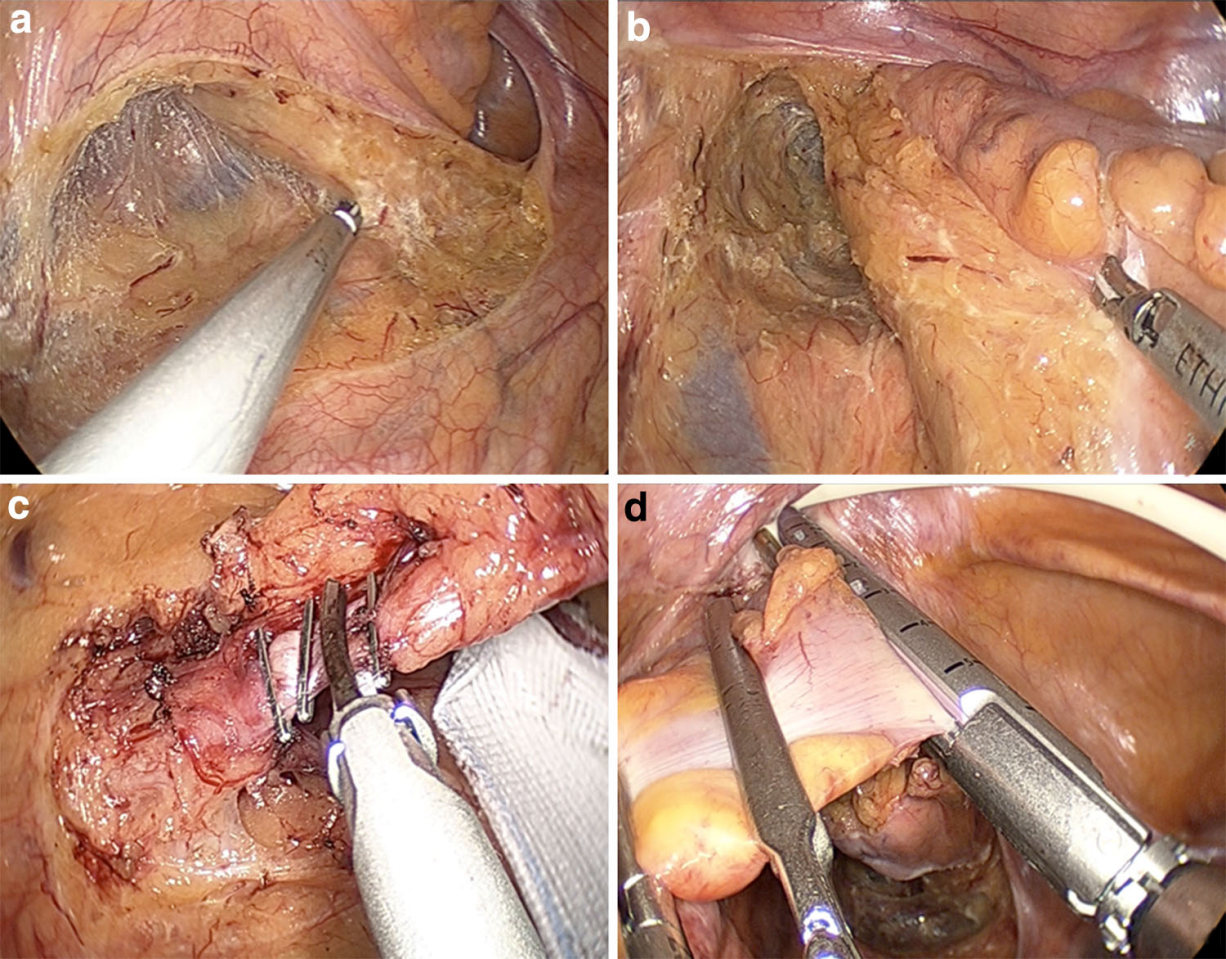
Operative techniques for single-site laparoscopic sigmoidectomy with complete mesocolic excision for sigmoid colon cancer. **A** Medial-to-lateral approach with initial peritoneal dissection near the promontorium. **B** Precise plane resection of the mesosigmoid by complete mesocolic excision. **C** Ligation at the origin of the inferior mesenteric artery with dissection of the entire mesosigmoid without injury to the nerves. **D** Intracorporeal transection of the rectum with an articulating linear stapler

The operations for left-sided colon cancer in both groups were performed via a traditional medial-to-lateral approach with the patient in the Trendelenburg position, as described previously [13] (Fig. 2A). After precise mesocolic resection with CME and partial mesorectal dissection in the TME plane (Fig. 2B), the inferior mesenteric artery was ligated and dissected between clips 0.5 cm from its aortic origin (Fig. 2C). The fat surrounding the rectum at least 5-cm distal to the lesion was removed, and the superior rectal vessels were dissected. The rectum was clamped for irrigation with saline from the anus and then transected intracorporeally by one firing of an articulating linear stapler (Fig. 2D). The specimen was extracted through the minilaparotomy incision in the umbilicus, and the double-stapling technique was applied for anastomosis.

The final incision was extended to a length comparable to the size of the specimen or the tumor. The wound was closed in layers, and the incision was remeasured. All patients were put under a similar enhanced postoperative care protocol. Intravenous narcotics were given as needed for postoperative pain control.

### Statistics

Data were collected and analyzed with the use of Microsoft Excel (Microsoft Corp., Redmond, WA, USA), and statistical calculations were performed with Prism 5.0 for Mac OS X (GraphPad Software, Inc., La Jolla, CA, USA). Between-group differences in variables were analyzed by means of the Chi square test or Student *t* test. A *p* value <0.05 was considered statistically significant.

## Results

Patient characteristics did not differ significantly between the SLC group and the MLC group (age, 64.3 ± 11.7 years vs. 65.6 ± 12.5 years, respectively, *p* = 0.353; male:female ratio (1.00 vs. 1.11, respectively, *p* = 0.644; BMI, 21.7 ± 3.3 vs. 22.4 ± 4.7 kg/m^2^, respectively, *p* = 0.137). No other clinical variables, i.e., ASA status, preoperative disease stage, and history of prior surgery, differed significantly between these two groups. In comparing these variables between the two groups on the basis of the tumor locations (left vs. right colon), no differences were found (Table 1).

Short-term outcomes (Table 2), including operation time, blood loss, number of lymph nodes harvested, and length of the resected specimen, were similar between the SLC group and the MLC group. The postoperative VAS pain score was significantly lower in the SLC group than in the MLC group (4.2 vs. 5.1; *p* = 0.01), but the pain scores did not differ significantly in relation to the side of the surgery. The postoperative complications are shown in Table 2. The overall complication rates were nearly equivalent in the two groups: (SLC, 12 % and MLC, 16.7 %; *p* = 0.249). There was no mortality or readmission within 30 days after the procedure in either group. Despite the lesser pain and similar short-term outcomes achieved with LCS, length of hospital stay did not differ significantly between the two groups (SLC, 8.2 days vs. MLC, 8.7 days; *p* = 0.152). The umbilical scars were almost invisible 3 months after the procedure, and almost all patients reported being very satisfied with the cosmetic outcomes.

**Table 2:** Short-term outcomes

	SLC-total (*n* = 150)	MLC-total (*n* = 150)		SLC-R (*n* = 69)	MLC-R (*n* = 69)		SLC-L (*n* = 81)	MLC-L (*n* = 81)	
			*p* value			*p* value			*p* value
Operation time (min)	172 ± 33	173 ± 35	0.720	168 ± 32	179 ± 32	0.046	174 ± 33	168 ± 37	0.21
Estimated blood loss (mL)	32 ± 26	37 ± 27	0.114	41 ± 32	46 ± 33	0.381	25 ± 16	29 ± 16	0.058
Length of initial skin incision (cm)	2.6 ± 0.5			2.7 ± 0.6			2.5 ± 0.4		
Length of final skin incision (cm)	3.0 ± 0.7	3.1 ± 1.0	0.317	3.2 ± 0.9	3.2 ± 1.2	0.912	2.8 ± 0.5	3.0 ± 0.8	0.058
Need for an enlarged incision	45 (30)			27 (39)	–		18 (22)		
Conversion to laparotomy	2 (1.3)	5 (3.3)	0.251	1 (1.4)	2 (2.9)		1 (1.1)	3 (3.7)	
Insertion of additional port(s)	12 (8.0)			3 (4.3)			9 (11.1)	–	
Postoperative VAS pain score	4.2 ± 2.7	5.1 ± 3.3	0.01	4.3 ± 3.0	5.3 ± 3.5	0.074	4.1 ± 2.4	4.9 ± 3.1	0.068
Length of hospital stay (days)	8.2 ± 2.7	8.7 ± 3.3	0.152	8.0 ± 2.3	8.5 ± 2.8	0.254	8.2 ± 3.1	8.9 ± 3.8	0.201
Complications	18 (12.0)	25 (16.7)	0.249	9 (13.0)	13 (18.8)	0.352	9 (11.1)	12 (14.8)	0.483
Wound infection	5	4		3	2		2	2	
Anastomotic leakage	2	2		0	0		2	2	
Anastomotic bleeding	2	4		2	3		0	1	
Ileus	6	8		3	5		3	3	
Thrombosis	0	1		0	0		0	1	
Urinary	1	2		0	1		1	1	
Cardiovascular	0	1		0	0		0	1	
Pneumonia	1	1		0	1		1	0	
Wound dehiscence	1	0		1	0		0	0	
Hernia	0	2		0	1		0	1	
Re-admission within 30 days after procedure	0	0	–	0	0	–	0	0	–
Mortality	0	0	–	0	0	–	0	0	–

Number (and percentage) of cases are shown unless otherwise indicated

*SLC* single site laparoscopic colectomy, *MLC* multiport laparoscopic colectomy, *L* left, *R* right

Operation time was significantly shorter in the group treated by right-sided SLC than in the group treated by right-sided MLC (168 ± 32 vs. 179 ± 32 min, respectively; *p* = 0.046), whereas estimated blood loss was similar between the two groups (41 ± 32 vs. 46 ± 34 mL, respectively; *p* = 0.381; Table 2). There was no difference in the number of lymph nodes harvested (23.9 vs. 23.7, respectively; *p* = 0.868) or the length of the resected specimen (22.3 vs. 22.3 cm; *p* = 0.991; Table 3). The right-sided SLC procedures were completed successfully except in four cases. Three patients required an additional port in the right lower quadrant due to visceral obesity or severe adhesion and the fourth required a small laparotomy for control of bleeding. The SLC procedure was completed without additional trocars in 94 % (65/69) of the right-sided cases; conversion to laparotomy was necessary in 1.4 % (1/69) of right-sided cases. Prolonged postoperative ileus developed in three patients, and anastomotic bleeding developed in two; no anastomotic leakage occurred (Table 2). The mean length of the final incision for a right-sided SLC was 3.2 cm; 27 patients (29 %) required extension of the original incision for gentle extraction of the tumor. Although the postoperative VAS pain score was slightly but not significantly lower for patients who underwent right-sided SCL than for those who underwent right-sided MLC (4.3 vs. 5.3; *p* = 0.074), length of hospital stay was similar between the two groups (8.0 vs. 8.5 days, respectively; *p* = 0.254; Table 2).

**Table 3:** Oncologic clearance

	SLC-total (*n* = 150)	MLC-total (*n* = 150)		SLC-R (*n* = 69)	MLC-R (*n* = 69)		SLC-L (*n* = 81)	MLC-L (*n* = 81)	
			*p* value			*p* value			*p* value
Number of lymph nodes harvested	22.2 ± 5.6	22.4 ± 6.0	0.767	23.9 ± 6.7	23.7 ± 7.4	0.868	20.7 ± 4.0	21.4 ± 4.4	0.291
Length of resected specimen (cm)	22.3 ± 5.1	21.6 ± 4.4	0.502	22.3 ± 5.4	22.3 ± 4.7	0.991	20.4 ± 4.7	21.1 ± 4.1	0.31
Tumor size (cm)	3.2 ± 1.4	3.3 ± 1.4	0.537	3.3 ± 1.3	3.4 ± 1.2	0.64	3.1 ± 1.5	3.2 ± 1.6	0.682

*SLC* single site laparoscopic colectomy, *MLC* multiport laparoscopic colectomy, *L* left, *R* right

All variables were similar between patients who underwent left-sided SLC and those who underwent left-sided MLC-L. Operation time (174 ± 33 vs. 167 ± 37 min, respectively; *p* = 0.21) and estimated blood loss (25 ± 16 vs. 29 ± 16 mL, respectively; *p* = 0.058) were similar (Table 2). There was no difference in the number of lymph nodes harvested (20.7 vs. 21.4, respectively; *p* = 0.291) or length of the resected specimen (20.4 vs. 21.1 cm, respectively; *p* = 0.31; Table 3). A distal tumor-free margin <5 cm was confirmed in all cases. The left-sided SLC procedure was completed in all but ten cases. Nine required an additional 12-mm trocar for insertion of a linear stapler for appropriate intracorporeal transection of the rectum or because of visceral obesity. There was only one conversion to open surgery, and this was due to severe adhesion. Successful completion and conversion rates were 88 % (71/81) and 1.1 % (1/81), respectively. Two patients developed a minor anastomotic leak, but the leaks were successfully managed conservatively without reoperation (Table 2). The mean final incision length in cases of left-sided SLC was 2.8 cm, and 18 (22 %) patients required further incision. The postoperative VAS pain score was slightly lower in the left-sided SCL group than in the left-sided MCL group (4.1 vs. 4.9; *p* = 0.068,), with similar hospital stays between groups (8.2 vs. 8.9 days; *p* = 0.201; Table 2).

In comparing right-sided SLC with left-sided SLC, the final skin incision was significantly longer (*p* = 0.008) and expansion of the initial incision was significantly more prevalent in the right-sided group than in the left-sided group (39 vs. 22 %, respectively; *p* = 0.024). In contrast, insertion of an additional port was slightly less prevalent in the right-sided group (4.3 vs. 11.1 %, respectively; *p* = 0.128), and operation time was slightly shorter in the right-sided group (168 vs. 174 min, respectively; *p* = 0.254). However, estimated blood loss was significantly greater in the right-sided group than in the left-sided group (41 vs. 25 mL, respectively; *p* < 0.001). Conversion to laparotomy and overall complication rates were nearly equivalent. No significant differences in any short-term outcomes were observed between the two surgeons who performed SLC.

## Discussion

Conventional laparoscopic surgery has achieved widespread acceptance as minimally invasive abdominal surgery, and its application to colorectal cancer has increased remarkably during the past decade [2–5]. However, each surgical wound required for conventional MLC may be a cause of postoperative pain and represent potential risk. Thus, even more minimally invasive techniques have been in recent demand. Surgeons experienced in conventional MLC are challenged to further decrease trauma and improve outcomes by reducing the number of ports and/or size of the trocars [23].

After SLC for colon cancer was introduced by Remizi et al. [24] and Bucher et al. [25] in 2008, the feasibility of the procedure was examined in two RCTs [21, 22] and in several case–control studies [14–20], which compared short-term outcomes between SLC and MLC. Although many authors have reported that SLC provides a better cosmetic result with similar perioperative results, the procedure remains somewhat controversial. Until now, with the exception of one report by Champagne et al. [20], most reports were based on limited data and a small number of selected cases. In addition, several studies of SLC were designed to include both cancerous and noncancerous lesions, such as adenoma, diverticulitis, or inflammatory disease [16–18, 20]. In the management of malignant lesions, certain oncologic clearance is the most important task. The manner by which to best dissect the regional lymph nodes or remove the mesocolon in SLC remains to be more carefully evaluated. To our knowledge, the present case–control study of SLC for colon cancer is the largest and also the first to examine SLC with CME.

Four case–control studies have been conducted to assess short-term outcomes of SLC [14, 15, 18, 20], but the results were controversial. Poon et al. conducted an RCT of SLC versus conventional laparoscopic colectomy in which postoperative pain was measured as the primary outcome variable; they reported reduced postoperative pain associated with a shorter hospital stay for patients treated by SLC [21]. Our finding that postoperative pain was greater in patients treated by MLC than in those treated by SLC corresponded to the findings that came out of the largest case–control study conducted [20] and one RCT [21]. This suggests that the lateral port sites in the abdominal wall contribute substantially to postoperative discomfort. However, reduced postoperative pain with similar perioperative outcomes (including complications) resulting from SLC was not enough to affect hospital stay in our patient series. This was largely due to our hospital’s discharge policy. It also might have been due to the fact that postoperative pain was evaluated only on POD 1. It remains unclear whether the reduced postoperative pain leads to faster postoperative recovery. The minimal invasiveness of SLC should be assessed and verified by detailed analysis of postoperative pain at all port sites in a future RCT.

The significantly longer final SLC incisions and the more frequent need for extending the length of the SLC incisions in our patients with cancers on the right versus the left were considered to be due to the volume of the extraction specimens. The extraction specimens tended to be greater volume in the right-sided group because of the loop formation with the double tract. In the left-sided group, there was a single tract with the transected stump of the distal colon.

Despite the technical difficulty of SLC, all but two studies, including two RCTs, reported similar operative times [18, 19]. The reported median SLC operation time ranges from 83 to 225 min [26], and the times are quite acceptable compared with the times for MLC [2–5]. Although the more careful and precise procedure that includes CME may necessitate a longer operation, our 168 min for right-sided colon cancer and 174 min for left-sided colon cancer are reasonable. Standardization of both MLC and SLC, whether on the right or the left, will make laparoscopic CME a reliable and safe procedure. Blood loss in our SLC cohort (25 mL in right-sided SLC and 41 mL in left-sided SLC) was slightly less than the losses previously reported. Although the level of difficulty may be increased for SLC with CME, it is possible to complete this precise procedure safely.

Interestingly, operation time was shorter in our right-sided SLC group than in our left-sided SLC group, and operation time was longer in our left-sided SLC group than in our left-sided MLC group. Conversion to open surgery occurred in only two SLC cases, and this number was remarkably lower than the five MLC cases requiring conversion. This could have been due to selection bias despite our every effort to match the cases. It also is possible that the performance of SLCs by well-experienced laparoscopic surgeons in carefully selected patients influenced the outcomes. The number of patients requiring an additional port was notably high when left-sided SLC was performed. This was due mainly to appropriate transection of the rectum. Even for standard laparoscopic surgery for rectal cancer, evaluation of technical and oncologic feasibility has just begun [27]. Thus, application of single-site laparoscopic surgery to rectal cancer should perhaps be selectively applied at present. It is reassuring that the surgeon can insert one or more additional trocars according to his own judgment at any time during the procedure. We also are reassured that our data showed the overall postoperative complication rate in SLS was nearly equivalent to that in MLC regardless of the side of the procedure, and there was no mortality.

With regard to oncologic clearance, in our SLC series with CME, the mean numbers of lymph nodes harvested (24 in right-sided cases and 21 in left-sided cases) were acceptable and comparable to previously reported numbers [9–12]. More than 12 lymph nodes were dissected in all cases except 3. The mean length of the resected specimen was also acceptable, with adequate tumor-free distal and proximal surgical margins. Oncologic resection with meticulous mesocolic dissection and optimal lymph node clearance may improve oncologic outcomes [9, 10]. The embryologic tissue planes must be respected to minimize the likelihood of cancer recurrence, and true central ligation of the lymphatic drainage maximizes regional lymph node harvest [11]. Standardization of CME has improved oncologic outcomes without increasing the postoperative complication or mortality rates [28]. During a median follow-up period of 24 months, 146 patients (97 %) who underwent SLC were free of recurrence (of the remaining 4 patients, 3 suffered liver metastasis and 1 suffered lung metastasis), and no local or lymph node recurrence was found.

Our study limitations should be noted. There likely were unmatched variables between the two groups, and these variables should be identified and addressed in future, randomized studies to reduce the potential selection bias. Furthermore, whether advanced colon cancer, transverse colon cancer, and rectal cancer are indicated for SLC should be evaluated as well as the long-term oncologic outcomes, the costs, training for SLC, and the stress levels of surgeons performing the procedure.

In conclusion, our study revealed that SLC with CME is feasible and safe when performed by experienced surgeons for selected patients. This procedure provides improved cosmesis and possible reduced postoperative pain with acceptable short-term outcomes and certain oncologic clearance. We hope that the short-term outcomes reported here will encourage future, prospective, randomized analysis to validate SLC with CME as a preferable alternative to conventional laparoscopy.
